# SARS-CoV-2 strains and clinical profiles of COVID-19 patients in a Southern Brazil hospital

**DOI:** 10.3389/fimmu.2024.1444620

**Published:** 2024-12-18

**Authors:** Bibiana S. de Oliveira Fam, Nathan Araujo Cadore, Renan Sbruzzi, Marilea Furtado Feira, Giovanna Câmara Giudicelli, Luiz G. P. de Almeida, Alexandra L. Gerber, Ana Paula de C. Guimarães, Ana Tereza Ribeiro Vasconcelos, Alexandre C. Pereira, Lygia V. Pereira, Tábita Hünemeier, Suzi Alves Camey, Fernanda S. Luiz Vianna

**Affiliations:** ^1^ Laboratory of Genomic Medicine, Center of Experimental Research, Hospital de Clínicas de Porto Alegre (HCPA), Porto Alegre, Brazil; ^2^ Laboratory of Immunobiology and Immunogenetics, Graduate Program in Genetics and Molecular Biology, Department of Genetics, Institute of Biosciences, Federal University of Rio Grande do Sul, Porto Alegre, Brazil; ^3^ Bioinformatics Core, Hospital de Clínicas de Porto Alegre (HCPA), Porto Alegre, Brazil; ^4^ Graduate Program in Genetics and Molecular Biology, Federal University of Rio Grande do Sul, Porto Alegre, Brazil; ^5^ Bioinformatics Laboratory, National Laboratory of Scientific Computation, LNCC, Petrópolis, Rio de Janeiro, Brazil; ^6^ Heart Institute, University of Sao Paulo Medical School, São Paulo, Brazil; ^7^ Department of Genetics and Evolutionary Biology, Institute of Biosciences, University of São Paulo, São Paulo, Brazil; ^8^ National Laboratory of Embryonic Stem Cells, University of Sao Paulo, São Paulo, Brazil; ^9^ Laboratory of Human Population Genomics, University of Sao Paulo, São Paulo, Brazil; ^10^ Statistics Department, Federal University of Rio Grande do Sul (UFRGS), Porto Alegre, Rio Grande do Sul, Brazil; ^11^ Statistics Department, Hospital de Clínicas de Porto Alegre (HCPA), Porto Alegre, Rio Grande do Sul, Brazil; ^12^ INAGEMP, National Institute of Populational Medical Genetics, Porto Alegre, Brazil

**Keywords:** Coronavirus disease, Coronavirus strains, clinical outcomes, healthcare, Brazilian population

## Abstract

**Introduction:**

The COVID-19 pandemic had a widespread global impact and presented numerous challenges. The emergence of SARS-CoV-2 variants has changed transmission rates and immune evasion, possibly impacting the severity. This study aims to investigate the impact of variants on clinical outcomes in southern Brazil.

**Methods:**

In total, samples from 277 patients, hospitalized and non-hospitalized, were collected between March 2020 and March 2021, before the vaccine was made widely available to the general population in Brazil. Whole genome sequencing of SARS-CoV-2 was performed and bioinformatics and biostatistics analyses were implemented on molecular and clinical data, respectively.

**Results:**

The study identified significant demographic and clinical differences. The hospitalized group exhibited a higher proportion of males (51.9%) and an increased prevalence of comorbidities, including hypertension (66.0%), obesity (42.6%), and chronic kidney disease (23.6%). Patients were identified with twelve SARS-CoV-2 strains, predominantly B.1.1.28 and B.1.1.33 in the early 2020 first wave, and P.1 overlapping in the late 2020 and early 2021 second wave of COVID-19. Significant differences in hospitalization rates were found among patients infected with the different SARS-CoV-2 lineages: B.1.1.33 (46.0%), B.1.1.28 (65.9%), and P.1 (97.9%). Severity markers, such as pneumonia (62.5%, p=0.002), acute respiratory distress syndrome (ARDS, 72.9%, p<0.001), and oxygen support >6 L/min O_2_ (64.6%, p<0.001), were more frequent in patients from the second wave. These findings highlight the impact of different variants on the clinical evolution and prognosis of COVID-19, especially when comparing the first and second waves of the pandemic.

**Conclusions:**

The study underscores the association between SARS-CoV-2 strains and COVID-19 severity by integrating clinical and viral data for public health responses during different pandemic phases, highlighting the importance of adapting pandemic strategies as the pandemic evolves.

## 1 Introduction

Severe Acute Respiratory Syndrome Coronavirus 2 (SARS-CoV-2), the virus responsible for COVID-19, has caused over 700 million infections and 7 million deaths worldwide (1). In Brazil, more than 37 million COVID-19 cases and 700,000 deaths have been reported, making it one of the countries most affected by the pandemic. Brazil ranks sixth in cumulative cases and second in cumulative deaths worldwide (https://COVID19.who.int/). The first confirmed case in the country was recorded in São Paulo in February 2020, followed by a rapid increase in transmissions and fatalities (2). Over time, multiple SARS-CoV-2 variants have been identified and co-circulated in the country, including the lineage B.1.1.28, first identified in Rio de Janeiro, in March 2020 (3, 4). This lineage, with mutations across the viral genome including a key mutation on the Spike protein’s S gene, spread rapidly throughout the country (3) and later gave rise to the variant of concern (VOC) P.1 and the variant of interest (VOI) P.2, known as Gamma and Zeta, respectively (5, 6). Another early variant, B.1.1.33, also featured Spike protein mutations in the receptor-binding domain (RBD), though it was less discussed. In late 2020, the emergence of the Gamma lineage in the Amazonas state, located in the North region of Brazil, coincided with an increasing overlap of this variant with other circulating strains (7). Characterized by core mutations that enhance the binding affinity to the human ACE2 receptor, this lineage rapidly spreads across the country and lead to a second epidemic wave with higher rates of infection, hospitalization, and death across the country (8).

The dynamics of the SARS-CoV-2 evolution and the independent introduction events of new variants have contributed to the emergence of additional VOIs and VOCs (9, 10). These evolving patterns were responsible for driving three distinct epidemic waves in Brazil, each marked by exponential increases in infections (11, 12), representing a significant challenge for the management of epidemiological containment measures. COVID-19 waves have been defined and monitored by different institutions worldwide (13), including in Brazil by the SIVEP-Gripe (Information System for Epidemiological Surveillance of Influenza) of the Ministry of Health, which uses hospitalization rates to delineate wave periods (opendatasus.saude.gov.br) (14). The first wave, from February 2020 to July 2020, was dominated by lineages B.1.1.28 and B.1.1.33 (5, 9, 11). The second wave, from November 2020 to December 2021, was the longest and most severe, characterized by the dominance of the Gamma variant (11). The third wave, from December 2021 to May 2022, was less severe and primarily driven by the Omicron variant. Studies suggest that certain VOIs and VOCs are associated with more severe clinical outcomes, including higher rates of hospitalizations, ICU admissions, and mortality (2). Lineages such as B.1.1.7 (Alpha), B.1.351 (Beta), B.1.617.2 (Delta), and P.1 (Gamma) have mutations that increase binding affinity, viral replication, and improve immune system evasion, leading to higher transmission rates and chances to reinfection (11, 13).

Key mutations within the SARS-CoV-2 genome, particularly in the Splike protein’s RBD, confer adaptive advantages to the virus (15, 16). These mutations among different strains, such as K417T, E484K, N501Y, and D614G, improve the virus’s ability to bind ACE2 receptor, alter immune response, and affect viral load (17–20). Additional mutations within other genomic regions have also been considered key factors in understanding the evolution of SARS-CoV-2 lineages and the course of the pandemic. For instance, the R246I in the N-terminal domain (NTD), a key point for human antibody recognition (21) plays a role in immune evasion and may impact vaccine and treatment effectiveness (16). Emerging evidence suggests that specific mutations and lineages can influence clinical outcomes, with variants such as B.1.1.7 and P.1 associated with increased disease severity and mortality in some populations (5, 22). For example, the P.1 variant, predominant in Brazil during certain periods, has been linked to higher viral loads and increased risk of severe COVID-19, possibly due to its enhanced transmissibility and immune evasion mechanisms (5, 23). Similarly, the Delta variant (B.1.617.2) has shown associations with higher hospitalization rates compared to earlier lineages (22, 24). Despite these insights, there is ongoing debate about the precise contribution of the lineages to the clinical outcomes of COVID-19 (25). Beyond viral genetics linked to more severe outcomes, other biological factors like age, gender, previous comorbidities, and host genetics also contribute to COVID-19 outcomes and severity (20). While studies have shown differences in hospitalization, pneumonia, and ICU admission due to infection from different VOCs of large circulation, such as B.1.1.17 and B.1.1.35 (26), others demonstrated that previous comorbidities are more critical in determining outcome than a specific mutation in the SARS-CoV-2 (27). Considering that the COVID-19 pandemic represented a great demand on the health system, this study aimed to investigate the characteristics of patients admitted to a reference assistance center in southern Brazil and assess the impact of different SARS-CoV-2 strains on clinical outcomes.

## 2 Methods

### 2.1 Sampling and ethical aspects

This cross-sectional study was conducted from March 2020 to March 2021 at the Hospital de Clínicas de Porto Alegre (HCPA) in Porto Alegre in southern Brazil. Hospital de Clinicas de Porto Alegre is a public tertiary-care hospital, which was a COVID-19 reference hospital, located at the city of Porto Alegre (1,488,000 inhabitants), the capital of Rio Grande do Sul State, Brazil. This hospital has treated patients from various regions of the city and the state of Rio Grande do Sul, which we believe enhances the representativeness of our sample, as seen in other publications. The vaccination against SARS-CoV-2 was not available for their ages or risk factors. All individuals had a positive RT-PCR for SARS-CoV-2, involving 200 participants with mild symptoms of COVID-19 and 300 participants with more severe outcomes. Samples of the positive nasopharyngeal–throat combined swabs were obtained for 277 patients from the Biobank of the HCPA (doi:10.22491/hcpa-biobanco-amostras). Sociodemographic and clinical data were retrieved retrospectively from medical records to analyze comorbidities, main symptoms during hospitalization, and the related outcome, including only those with clinical data and SARS-CoV-2 lineage sequencing. Epidemiological data for the municipality of Porto Alegre were obtained from the Health Department of the State of Rio Grande do Sul (https://ti.saude.rs.gov.br/covid19/). The data include the distribution of SARS-CoV-2 lineages and the corresponding epidemiological curve. This study was approved by the Research Ethics Committee of HCPA (CAAE: 36974620.3.0000.5327).

### 2.2 Nucleic acid isolation and RT–qPCR

SARS-CoV-2 RNA samples from nasopharyngeal–throat swabs were processed by the QIAamp^®^ Viral RNA Mini Kit as recommended by the supplier. Reverse transcription was performed using the kit SuperScript™ IV First-Strand Synthesis System (Invitrogen) according to the manufacturer’s guidelines. The cDNA was used in two multiplex PCR assays using the Q5^®^ High-Fidelity DNA Polymerase and a primer scheme from a standard protocol (https://www.genomahcov.fiocruz.br/). PCR products were purified using Agencourt AMPure XP beads (Beckman Coulter™), and the DNA concentration was measured by the Qubit Kit Fluorometer (Invitrogen) using the Qubit dsDNA HS Assay Kit (Invitrogen). DNA Multiplex products were normalized and pooled together in a final concentration of 50 pmol. Only samples presenting Ct values ≤28 were considered for genome viral sequencing.

### 2.3 Library construction and sequencing

Viral libraries were prepared using the Illumina COVIDSeq Test (Illumina) according to the manufacturer’s protocol at the DFA/LNCC Genomics Unit. The amount of 8.5 ul of previously extracted viral RNA from each sample was used as input for library construction. Subsequently, 5 ul aliquots of each library were combined in pools of 96 libraries, and then purified. Each pool was analyzed using the TapeStation System (Agilent) for quality control and quantification. One NextSeq 500/550 Mid Output Kit v2.5 (300 Cycles) was used to generate reads of 2×149 bp in the NextSeq 500 System (Illumina). The Illumina DRAGEN COVID Lineage v3.5.1 pipeline was used for comprehensive sequence analysis, consensus building, and variant calling.

### 2.4 Statistical analysis

The characteristics of the study sample were evaluated according to clinical and sociodemographic data based on the medical records related to each of the individuals included in the study. A comprehensive descriptive analysis was conducted to summarize the distribution of these variables, offering an overview of frequency patterns. Pearson’s chi-square test was used to evaluate the potential association between sociodemographic and clinical variables with SARS-Cov-2 lineages. This test was chosen as it is appropriate for assessing the relationships between categorical variables, allowing us to determine if differences in characteristics are significantly associated with specific lineages. In cases where expected frequencies were low, Fisher’s exact test was used as an alternative to Pearson’s chi-square to ensure the robustness of the results. We also employed appropriate adjustments for multiple comparisons where applicable, including the Bonferroni correction, to control the risk of Type I errors. Logistic regression models are also implemented to explore associations between SARS-CoV-2 strains, specifically viral strains, P.1., B.1.1.28 and B.1.1.33, and severity markers outcomes such as the presence of ARDS, pneumonia, ICU admission, and the need for oxygen >6L. The models were adjusted for potential confounders, including age at infection, hypertension, and obesity. Four logistic regression models were conducted. Odds Ratios (ORs) with 95% Confidence Intervals (CIs) were calculated for each model to estimate the likelihood of each outcome, and p-values indicated the statistical significance of the associations. Analyzes were performed using the SPSS Statistics v27.0 software (Armonk, NY: IBM Corp) and the R software (R Core Team, 2022).

## 3 Results

In total, viral samples from 277 participants were sequenced, 162 (58.5%) from patients hospitalized due to COVID-19 and 115 (41.5%) from non-hospitalized individuals. A higher proportion of men were found in the hospitalized group, 84 individuals (51.9%), compared to the non-hospitalized group with 35 individuals (30.4%) (*p* = 0.001). Among hospitalized patients, a higher proportion of individuals with obesity (42.6%) as well as other comorbidities such as chronic kidney disease (CKD, 24.1%), diabetes (51.2%), hypertension (66.0%), other chronic diseases (59.9%) and immunodeficiencies (11.1%) (**Table 1**). Pneumonia was a prevalent complication in this group, affecting 114 (70.4%) patients. Moreover, 95 (58.6%) patients presented acute respiratory distress syndrome (ARDS) and 76 (46.9%) required admission to the ICU. Oxygen supplies with less than 6 L/min O_2_ were required in 37 patients (13.0%) and more than 6 L/min O_2_ in 96 patients (35.0%). Other complications such as kidney injury (22.0%), cardiovascular complications (8.3%), hepatic injury (2.9%), and venous thromboembolism (11.6%) were observed in admitted patients (**Table 1**).

**Table 1:** Clinical characteristics and frequencies within the COVID-19 study cohort with statistically significant differences at the *p* < 0.05 level (*CKD- Chronic kidney disease, **ARDS- Acute Respiratory Distress Syndrome, **ICU- Intensive care unit) (*n*=277).

	Hospitalized, n (%)	Non-hospitalized, n (%)	Total, n (%)	*p-*value
**Male**	84 (51.9)	35 (30.4)	119 (43.0)	0.001
**White**	136 (84.0)	96 (83.5)	232 (83.8)	0.007
**Age, median (min-max)**	63 (19-102)	40 (25-74)	52 (19-102)	-
**Obesity**	69 (42.6)	31 (27)	100 (36.1)	0.008
**Chronic diseases**	97 (59.9)	41 (35.7)	138 (49.8)	<0.001
CKD*	39 (24.1)	0 (0.0)	39 (14.1)	<0.001
**Diabetes**	83 (51.2)	2 (1.7)	85 (30.7)	<0.001
**Hypertension**	107 (66.0)	25 (21.7)	132 (47.7)	<0.001
**Immunodeficiencies**	18 (11.1)	0 (0)	18 (6.5)	<0.001
**Co-infections**	89 (54.9)	0 (0)	89 (32.1)	<0.001
**Pneumonia**	114 (70,4)	0 (0)	114 (70,4)	<0.001
ARDS**	95 (58.6)	0 (0)	95 (58.6)	<0.001
**ICU**	76 (46.9)	0 (0)	76 (46.9)	<0.001
**Oxygen (<6LO_2_)**	37 (66.7)	0 (0)	37 (66.7)	<0.001
**Oxygen (>6LO_2_)**	96 (59.3)	0 (0)	96 (59.3)	<0.001
**Kidney Injury**	61 (22)	0 (0)	61 (22)	<0.001
**Cardiovascular Complications**	23 (8.3)	0 (0)	23 (8.3)	<0.001
**Hepatic Injury**	8 (2.9)	0 (0)	8 (2.9)	<0.001
**Venous Thromboembolism**	32 (11.6)	0 (0)	32 (11.6)	<0.001
**Survival**	91 (56.2)	115 (100.0)	206 (74.4)	<0.001
**Total**	162 (58.5)	115 (41.5)	277 (100)	-

Bold formatting highlights statistically significant values (p < 0.05).

**Figure 1:**
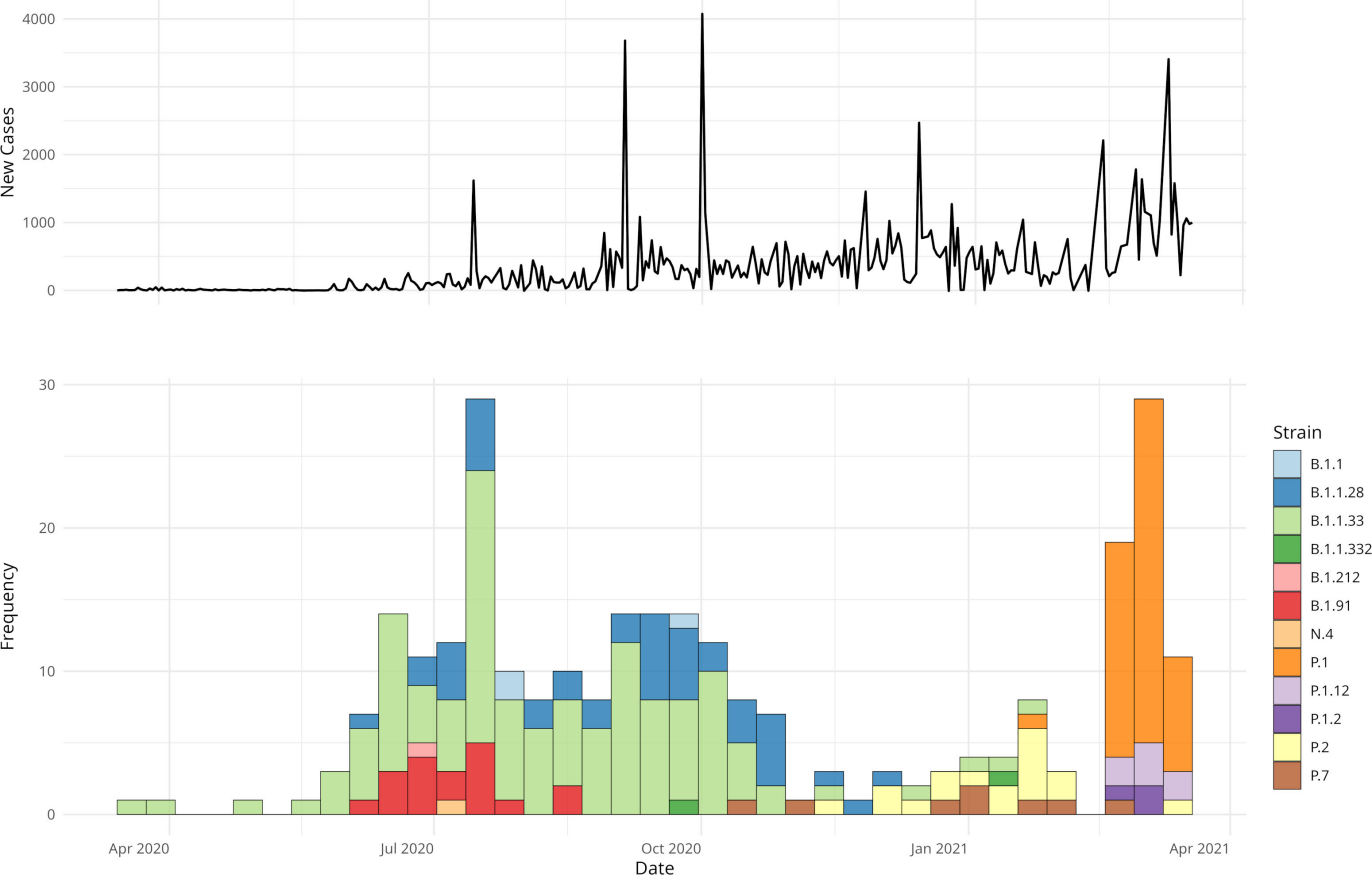
COVID-19 trends in Porto Alegre and in hospital strain distribution. The upper panel shows an epidemiological curve referent to the daily new number of cases in the city, highlighting outbreak dynamics. The lower panel displays the frequency of HCPA strain samples, grouped by 7-day intervals and colored by type.

Twelve different lineages were identified, circulating from March 2020 to March 2021 (**Figure 1**; **Table 2**). Among these, three lineages were predominant: B.1.1.28, identified in 44 individuals (18.0%); B.1.1.33, identified in 124 individuals (35.4%); and P.1, identified in 48 individuals (29.2%). Together, these three lineages accounted for 78.0% of all cases. The data also reflect two distinct phases of the pandemic (**Figure 1**). In addition to the lineage distribution, **Figure 1** includes the epidemiological curve corresponding to the COVID-19 incidence data for the municipality of Porto Alegre, demonstrating the peaks of case occurrence during the first and second waves of the pandemic. During the early months of 2020, the first wave of COVID-19 was characterized by a predominance of B.1.1.28 and B.1.1.33 strains. From November 2020, the second wave emerged, marked by a rapid increase in the prevalence of the Gamma variant, which overlapped with other circulating strains. In early 2021 (January-February), a decline in the prevalence of the lineage B.1.1.33 was observed, while the P.1 lineage became more predominant. Furthermore, sub-lineages of Gamma, specifically P.1.12 and P.1.2, were identified in 8 and 3 individuals, respectively. Moreover, two other strains, P.2 and P.7, were also observed in this period.

**Table 2:** Distribution of different strains of the virus (SARS-CoV-2) based on hospitalization within the COVID-19 study cohort.

Strain	Hospitalized, n (%)	Non-hospitalized, n (%)	total, n (%)
**B.1.1**	1 (0.6)	2 (1.7)	3 (1.1)
**B.1.1.28,**	29 (17.9)	15 (13)	44 (15.9)
**B.1.1.33,**	57 (35.2)	67 (58.3)	124 (44.8)
**B.1.1.332**	1 (0.6)	1 (0.9)	2 (0.7)
**B.1.212**	1 (0.6)	0 (0)	1 (0.4)
**B.1.91**	4 (2.5)	14 (12.2)	18 (6.5)
**N.4,**	1 (0.6)	0 (0)	1 (0.4)
**P.1**	48 (29.6)	0 (0)	48 (17.3)
**P.1.12**	8 (4.9)	0 (0)	8 (2.9)
**P.1.2**	2 (1.2)	1 (0.9)	3 (1.1)
**P.2,**	7 (4.3)	10 (8.7)	17 (6.1)
**P.7**	3 (1.9)	5 (4.3)	8 (2.9)
**Total**	162 (100)	115 (100)	277 (100)

Bold formatting highlights statistically significant values (p < 0.05).

A comprehensive comparison of clinical characteristics and outcomes in patients infected with three dominant strains (B.1.1.28, B.1.1.33, and P.1) revealed no statistically significant associations between demographic factors, such as sex and ethnicity, and the strain of infection (**Table 3**). However, the median age varied across the lineages with patients infected with the P.1 variant having the highest median age (64 years), followed by B.1.1.33 (63.5 years), and B.1.1.28 (57.2 years). The prevalence of comorbidities also varied by lineage, with hypertension being more common in P.1 (66.7%, 32 individuals), followed by B.1.1.33 (40.3%, 50 individuals) and B.1.1.28 (52.3%, 24 individuals) infected patients (p = 0.007, **Table 3**). Specific medical complications were found to be associated with different SARS-CoV-2 strain infections. For instance, P.1 strain infection was associated with a higher ICU admission rate, with 20 patients requiring intensive care (41.7%, p=0.01, **Table 3**). In addition, individuals infected with P.1 had a significantly higher incidence of pneumonia (62.5%, p=0.002) than individuals compared to those infected with B.1.1.28 (47.4%) and B.1.1.33 (33.1%). P.1 infection was also linked to a higher incidence of ARDS in 72.9% of patients, compared to 36.4% in B.1.1.33 and 21% in B.1.1.28 infections (p<0.001, **Table 3**). Furthermore, the need for a high flow oxygen supply (>6 L/min O_2_) was more common in individuals infected with the P.1 strain (64.6%) compared to those infected with B1.1.28 (36.4%) and B.1.1.33 (27.4%) strains. Individuals infected with P.1 also had a higher rate of kidney injury (37.5%) than those infected with B.1.1.28 (27.3%) and B.1.1.33 (18.5%) lineages (p=0.031, **Table 3**).

**Table 3:** Clinical profile of patients admitted due to Covid-19 sampled in the present study from March 2020 to March 2021, stratified by SARS-CoV-2 strain (*ICU- Intensive care unit, **ARDS-Acute Respiratory Distress Syndrome) (*n=216*).

	B.1.1.28, *n* (%)	B.1.1.33, *n* (%)	P.1, *n* (%)	Total, *n* (%)	*p*
**Male**	23 (52.3)	56 (45.2)	22 (45.8)	101 (46.8)	0.710
**White**	37 (84.1)	105 (84.7)	38 (79.2)	180 (83.3)	0.208
**Age med (max-min)**	52.77 (27-84)	51.37 (25-102)	63 (19-92)	51.0 (51.8-64.0)	–
**Hypertension**	23 (52.3)	50 (40.3)	32 (66.7)	105 (48.6)	**0.007**
**Obesity**	16 (36.4)	40 (32.3)	18 (37.5)	74 (34.3)	0.767
**Diabetes**	16 (36.4)	33 (26.6)	22 (45.8)	71 (32.9)	**0.047**
**Hospitalization**	29 (65.9)	57 (46)	47 (97.9)	133 (61.7)	**<0.001**
**Pneumonia**	21 (47.7)	41 (33.1)	30 (62.5)	92 (42.6)	**0.002**
ARDS*	16 (36.4)	26 (21)	35 (72.9)	77 (35.6)	**<0.001**
ICU**	14 (31.8)	25 (20.2)	20 (41.7)	59 (27.3)	**0.013**
**Kidney Injury**	12 (27.3)	23 (18.5)	18 (37.5)	53 (24.5)	**0.031**
**Cardiovascular complications**	4 (9.1)	8 (6.5)	8 (16.7)	20 (9.3)	0.117
**Venous Thromboembolism**	2 (4.5)	16 (12.9)	7 (14.6)	25 (11.6)	0.251
**Oxygen support >6L/min**	16 (36.4)	34 (27.4)	31 (64.6)	81 (37.5)	**<0.001**
**Deceased**	7 (15.9)	28 (22.6)	20 (41.7)	55 (25.5)	**<0.001**

Bold values indicate statistical significance (p < 0.05).

The results of the analysis of risk associated with SARS-CoV-2 lineages highlight significant differences in clinical outcomes, particularly concerning the development of ARDS and the need for supplemental oxygen (**Supplementary Table S1**). The study revealed that the P.1 lineage was significantly associated with an increased risk of developing Acute Respiratory Distress Syndrome (ARDS), with an odds ratio (OR) of f 7.831 (95% CI: 3.427–18.886; p < 0.001). Additionally, the P.1 lineage was also associated with an increased risk of requiring more than 6 liters of oxygen (OR = 2.906, 95% CI: 1.287–6.693; p = 0.011). This increased risk remains significant even after controlling for confounding variables such as age, hypertension, and obesity, which have consistently been associated with severe outcomes in COVID-19. These results underline the critical role of the SARS-CoV-2 lineage in determining the severity of the disease and the need for targeted clinical interventions for specific variants. However, we did not find differences in the rates of cardiovascular complications or thromboembolism. Finally, there was a statistical association between death and variant strains, with P.1 exhibiting the highest death rate (41.7%), followed by B.1.1.33 (22.6%), and B.1.1.28 (15.9%) (p<0.001, **Table 3**). Overall, the estimated lethality rate among hospitalized patients was 38.5%.

## 4 Discussion

During an epidemic period, changes in viral strains are expected as the virus evolves and disperses over time across different geographic regions and between populations (28). Genomics surveillance is crucial for preparing for the emergence of new strains and mitigating the impact of outbreaks (26, 29, 30). As well as other countries, due to the COVID-19 pandemic Brazil faced a public health crisis with the rapid spread of the virus across the country, with large reports of cases and deaths (26). The country’s healthcare system structure has been tested, with many hospitals and ICUs overcrowded and a lack of medical resources, including oxygen. SARS-CoV-2, over the pandemic course, has shown itself to be a virus with a middle-low lethality rate (~2%) (28). However, it exhibits a high transmission rate and virulence, which led to reaching a large portion of the population in a short period, causing the collapse of health systems (31). Thus, in a dynamic scenario such as the COVID-19 pandemic, it is essential to understand the interaction between host and pathogen in response to different selection pressures (20).

**Figure 2:**
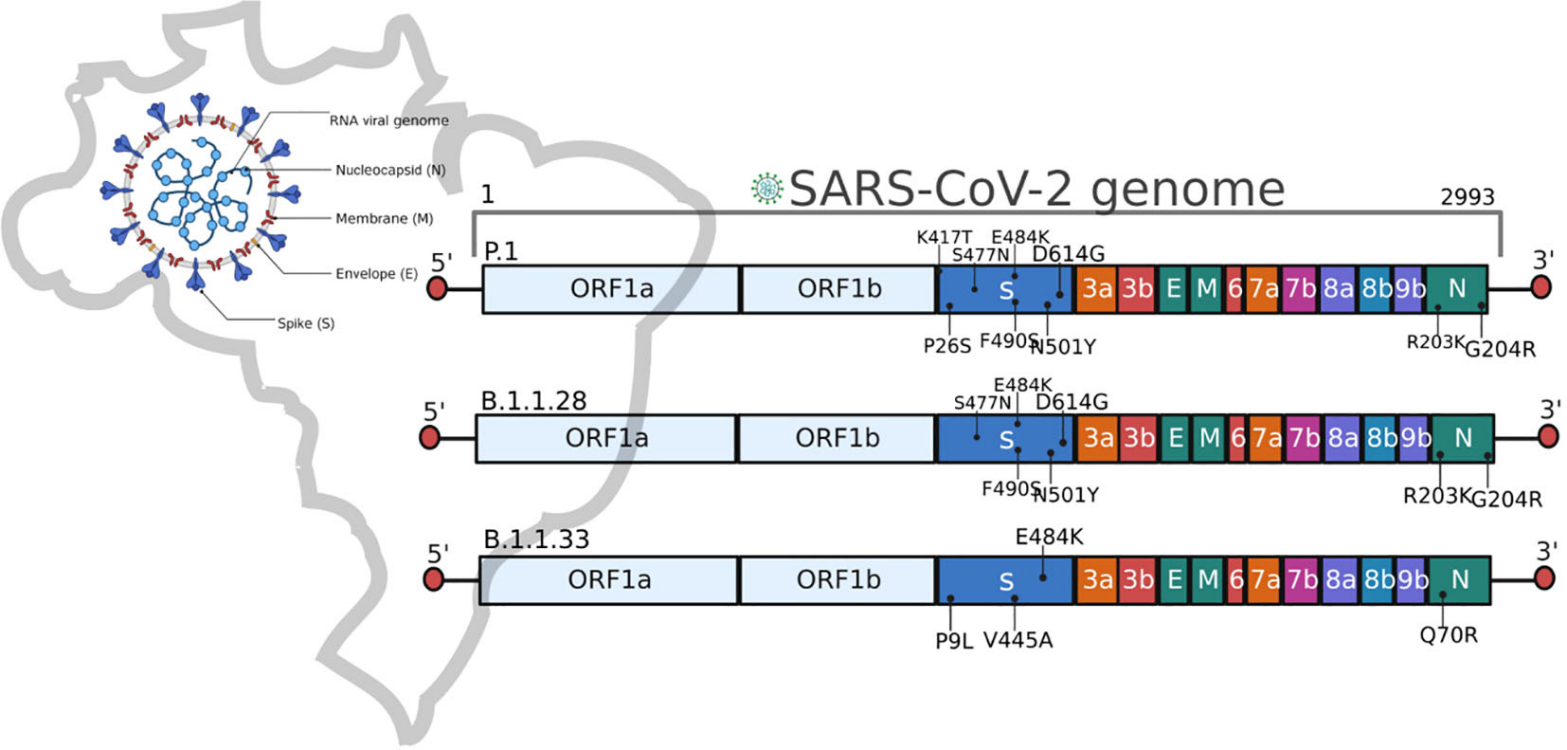
Schematic representation of the SARS-CoV-2 genome and the main mutations described in the Brazilian lineages B.1.1.28, B.1.1.33, and P.1.

Here, we identify that the main variants of SARS-CoV-2 sampled in the first wave, the B.1.128 and B.1.1.33 strains had significant clinical impacts on admitted individuals. Moreover, individuals infected with the B.1.1.28 lineage had higher rates of hospitalization, ICU admission, pneumonia, and ARDS than compared to B.1.1.33. Due to relevant mutations from a functional point of view (**Figure 2**), B.1.1.28 shares more than 10 mutations with P.1, including E484K in the RBD, impacting immune escape and response to therapies (28, 29). Brazil’s second wave of COVID-19 was notably more aggressive, with regional differences in infection and mortality (11, 20, 30). Although age and previous comorbidity as obesity are well-established risk factors for severe outcomes in COVID-19, our results highlight the importance of SARS-CoV-2 variants in the severity markers of clinical manifestations (32, 33). The P.1 lineage presented an increased risk of severe complications, such as ARDS and the need for oxygen, even after adjusting for confounding variables. This finding suggests that this variant may have intrinsic characteristics that increase its pathogenic potential, warranting greater attention in the clinical management of infected patients. In this specific period, the country experienced a higher in-hospital mortality, especially in older patients and those with comorbidities (30, 34), which aligns with our findings. Among hospitalized patients with severe COVID-19 symptoms, we observed high rates of associated comorbidities, particularly in individuals aged over 50 years.

The P.1 lineage shows critical mutations (**Figure 2**) that significantly affected the second wave by enhancing binding affinity to the human ACE2 receptor and increasing of transmission rate (17, 21, 35). In addition to viral mutations, other factors have been associated with protection or susceptibility to SARS-CoV-2 infection (36). However, as observed in other studies, here we report a higher rate of ICU admission, pneumonia, and ARDS incidence in the second wave of COVID-19 (2, 37, 38). Also, a consistently high comorbidity rate in hospitalized patients, which follows Zeister et al. (30), who described 56% of hospitalized Brazilian patients with comorbidities. The overall in-hospital mortality rates were 38.3%, similar to previously described by Peres et al. (34), which reported an in-hospital mortality of 37% in Brazilian patients. Throughout the pandemic course, in-hospital mortality increased from 34.8% in the first wave to 39.3% in the second wave (30). Besides, it is important to note that Brazil’s vaccination against COVID-19 began in January 2021 (34), with an initial focus on risk groups, emphasizing then that the data describes a critical period before the immunization of the general population. All participants of this study were not vaccinated at the moment of the SARS-CoV-2 infection described here.

This cross-sectional study has some limitations inherent to the study design, such as the use of convenience sampling available from the HCPA Biobank repository, which may not fully represent the epidemiological scenario with broad population coverage. Despite these limitations, the study results suggest a possible association between the P.1 variant of SARS-CoV-2 and the change in epidemiological profile in southern Brazil as suggested in other studies (14, 23, 30). Overall, this data highlights significant differences in the prevalence of chronic diseases, specific complications, ICU admissions, and death rates among the different strains. In a country of continental proportions with a wide ecological and sociodemographic diversity among its different regions, confronting the COVID-19 pandemic requires strategic planning to promote effective surveillance and sanitary measures. Determining the importance of different viral lineages and the implication in the clinical characteristics results in the improvement of future effective epidemiological surveillance and viral containment (39, 40). Furthermore, the arms race between the host-pathogen and its coevolutionary process can be fundamental in implementing new diagnoses, developing new therapies, and updating immunizers.

## Data Availability

A full list of the sequences employed in this work is available on GISAID under EPI_SET_231117yn (doi: 10.55876/gis8.231117yn). These sequences have also been deposited in GenBank under the accession numbers PP902194 - PP902359.
