# Supplementary material for: SARS-CoV-2 strains and clinical profiles of COVID-19 patients in a Southern Brazil hospital
Source: Front Immunol. 2024 Dec 18;15:1444620. doi: 10.3389/fimmu.2024.1444620 (PMC11688617; doi:10.3389/fimmu.2024.1444620)
Supplement: Supplementary file 1 [file Table1.docx]

**SARS-CoV-2 strains and clinical profiles of COVID-19 patients in a Southern Brazil hospital**

- ***Supplementary information -***

**Table S1.** Results of logistic regression models, adjusted for severity markers on clinical outcomes including ARDS, pneumonia, ICU admission, and need for oxygen >6L, are presented. The models include SARS-CoV-2 strains (P.1, B.1.1.28, and StrainB.1.1.33) and clinical parameters (age at infection, hypertension, and obesity) as potential confounders. For each variable, estimated coefficients (Estimate), standard errors (SE), Z-values (Z-Value), and p-values (p-value) are provided.

|  | **Variable** | **Estimate** | **Standard Error** | **Z-Value** | **p-value** | **OR** | **95% CI (Lower, Upper)** | **p-value (OR)** |
| --- | --- | --- | --- | --- | --- | --- | --- | --- |
| **ARDS** | Strain B.1.1.33 | -4.648 | 0.792 | -5.869 | 0.000 | 0.010 | (0.002, 0.042) | 0.000 |
|  | Strain B.1.1.28 | 0.818 | 0.418 | 1.957 | 0.050 | 2.266 | (0.994, 5.164) | 0.050 |
|  | Strain P.1 | 2.058 | 0.433 | 4.752 | 0.000 | 7.831 | (3.427, 18.886) | 0.000 |
|  | Age at infection | 0.048 | 0.013 | 3.756 | 0.000 | 1.049 | (1.024, 1.076) | 0.000 |
|  | Hypertension | 0.379 | 0.396 | 0.957 | 0.338 | 1.461 | (0.669, 3.182) | 0.338 |
|  | Obesity | 1.246 | 0.391 | 3.183 | 0.001 | 3.476 | (1.639, 7.662) | 0.001 |
| **Pneumonia** | Strain B.1.1.33 | -3.377 | 0.642 | -5.260 | 0.000 | 0.034 | (0.009, 0.114) | 0.000 |
|  | Strain B.1.1.28 | 0.593 | 0.381 | 1.555 | 0.120 | 1.809 | (0.856, 3.836) | 0.120 |
|  | Strain P.1 | 0.730 | 0.389 | 1.879 | 0.060 | 2.076 | (0.971, 4.482) | 0.060 |
|  | Age at infection | 0.044 | 0.011 | 3.825 | 0.000 | 1.045 | (1.023, 1.069) | 0.000 |
|  | Hypertension | 0.284 | 0.359 | 0.790 | 0.429 | 1.328 | (0.651, 2.674) | 0.429 |
|  | Obesity | 0.623 | 0.340 | 1.835 | 0.066 | 1.865 | (0.963, 3.662) | 0.066 |
| **ICU** | Strain B.1.1.33 | -3.533 | 0.730 | -4.844 | 0.000 | 0.029 | (0.006, 0.115) | 0.000 |
|  | Strain B.1.1.28 | 0.553 | 0.420 | 1.319 | 0.187 | 1.739 | (0.754, 3.945) | 0.187 |
|  | Strain P.1 | 0.560 | 0.404 | 1.387 | 0.166 | 1.751 | (0.788, 3.863) | 0.166 |
|  | Age at infection | 0.026 | 0.012 | 2.153 | 0.031 | 1.027 | (1.003, 1.052) | 0.031 |
|  | Hypertension | 1.075 | 0.404 | 2.663 | 0.008 | 2.931 | (1.348, 6.621) | 0.008 |
|  | Obesity | 0.419 | 0.364 | 1.152 | 0.250 | 1.520 | (0.744, 3.113) | 0.250 |
| **O2 > 6L** | Strain B.1.1.33 | -5.122 | 0.797 | -6.430 | 0.000 | 0.006 | (0.001, 0.026) | 0.000 |
|  | Strain B.1.1.28 | 0.427 | 0.417 | 1.025 | 0.305 | 1.533 | (0.672, 3.470) | 0.305 |
|  | Strain P.1 | 1.067 | 0.419 | 2.549 | 0.011 | 2.906 | (1.287, 6.693) | 0.011 |
|  | Age at infection | 0.066 | 0.013 | 4.993 | 0.000 | 1.068 | (1.042, 1.098) | 0.000 |
|  | Hypertension | 0.233 | 0.386 | 0.603 | 0.546 | 1.262 | (0.585, 2.679) | 0.546 |
|  | Obesity | 1.086 | 0.381 | 2.849 | 0.004 | 2.962 | (1.396, 6.367) | 0.004 |
